# Investigating Neurometabolite Changes in Response to Median Nerve Stimulation

**DOI:** 10.1002/brb3.70250

**Published:** 2025-01-08

**Authors:** Mairi S. Houlgreave, Katherine Dyke, Adam Berrington, Stephen R. Jackson

**Affiliations:** ^1^ School of Psychology University of Nottingham University Park Nottingham UK; ^2^ Sir Peter Mansfield Imaging Centre, School of Physics and Astronomy University of Nottingham University Park Nottingham UK; ^3^ Institute of Mental Health, School of Medicine University of Nottingham University Park Nottingham UK

**Keywords:** GABA, glutamate, magnetic resonance spectroscopy, median nerve stimulation, sensorimotor cortex

## Abstract

**Background:**

Rhythmic median nerve stimulation (MNS) at 10 Hz has been shown to cause a substantial reduction in tic frequency in individuals with Tourette syndrome. The mechanism of action is currently unknown but is hypothesized to involve entrainment of oscillations within the sensorimotor cortex.

**Objective:**

We used functional magnetic resonance spectroscopy (fMRS) to explore the dynamic effects of MNS on neurometabolite concentrations.

**Methods:**

Here, we investigated the effects of rhythmic and arrhythmic 10 Hz MNS on glutamate (Glu) and GABA concentrations in the contralateral sensorimotor cortex in 15 healthy controls, using a blocked fMRS design. We used a Mescher–Garwood‐semi‐localized by adiabatic selective refocusing (MEGA‐sLASER) sequence at 7 T.

**Results:**

Our results show no difference in the difference‐from‐baseline measures between the two stimulation conditions. Looking at the effect of MNS over both conditions there is a trend for an initial increase in Glu/tCr (total creatine) followed by a decrease over time, whereas GABA/tCr decreased during each stimulation block.

**Conclusions:**

These results suggest that despite entrainment of oscillations during rhythmic MNS, there are no significant differences in the tonic neuromodulatory effects of rhythmic and arrhythmic stimulation. The reduction in Glu over the course of stimulation may reflect a decrease in the glutamatergic firing due to adaptation. This may make it less likely that an involuntary movement is generated during continuous stimulation.

## Graphical abstract

There are no significant differences in the tonic neuromodulatory effects of rhythmic and arrhythmic stimulation. The reduction in glutamate over the course of stimulation may reflect a decrease in glutamatergic firing due to adaptation. This may make it less likely that an involuntary movement is generated during continuous stimulation.

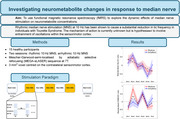

**Abbreviations**
Cho: choline
CRLB: Cramér–Rao lower bound
CSF: cerebrospinal fluid
DIFF: difference spectra
fMRI: functional magnetic resonance imaging
fMRS: functional magnetic resonance spectroscopy
GABA: gamma‐aminobutyric acid
GLM: general linear model
Gln: glutamine
Glu: glutamate
GM: gray matter
MEG: magnetoencephalography
MEGA‐sLASER: Mescher–Garwood‐semi‐localized by adiabatic selective refocusing
MNS: median nerve stimulation
NAA: *N*‐acetylaspartate
S1: primary somatosensory cortex
sem: standard error of the mean
SNR: signal‐to‐noise ratio
tCr: total creatine
TMS: transcranial magnetic stimulation
VAPOR: variable pulse power and optimized relaxation delay
Vmax: maximum compliance voltage
WM: white matter

## 1 Introduction

Rhythmic median nerve stimulation (MNS) has been shown to result in frequency specific increases in the amplitude and phase synchronization of neural oscillations (Houlgreave et al. 2022; Morera Maiquez et al. 2020). This modulation is restricted to the contralateral sensorimotor cortex and is not seen during arrhythmic stimulation (Houlgreave et al. 2022; Morera Maiquez et al. 2020). The mechanisms underpinning rhythmic MNS are of therapeutic interest as compared to periods of no stimulation, rhythmic application of 10 Hz MNS has also been demonstrated to produce substantial reduction in tic frequency and the urge‐to‐tic in individuals with Tourette syndrome (Iverson, Arbuckle, Song, et al. 2023; Iverson, Arbuckle, Ueda, et al. 2023; Maiquez et al. 2023; Morera Maiquez et al. 2020). In contrast to other non‐invasive stimulation methods, specifically transcranial magnetic stimulation (TMS), MNS offers an attractive approach to modulating brain sensorimotor networks implicated in brain health conditions such as Tourette syndrome, which could easily be adapted into a wearable therapeutic device.

Functional magnetic resonance imaging (fMRI) studies have shown that both unilateral movements and rhythmic MNS result in activation of cortical sensorimotor regions. During unilateral movements, there is activation in the contralateral sensorimotor cortex and deactivation of the ipsilateral sensorimotor cortex (Allison et al. 2000). Similarly, MNS at low frequencies (0.5–4 Hz) leads to the activation of the contralateral primary sensory cortex, bilateral secondary sensory cortex, and the bilateral insula (Backes et al. 2000; Ferretti et al. 2007; Manganotti et al. 2009). Furthermore, primary somatosensory cortex (S1) activation has been demonstrated to increase with stimulation frequency, although this increase plateaus at 10 Hz (Ferretti et al. 2007; Kampe, Jones, and Auer 2000; Manganotti et al. 2009).

Chen et al. (2017) used an fMRI localizer task to identify a voxel located in the motor cortex which was activated by a simple hand‐clenching task. Then, using functional magnetic resonance spectroscopy (fMRS), they demonstrated a significant increase in glutamate (Glu) and glutamine (Gln) within this voxel during the same task (Chen et al. 2017). Other fMRS studies have reported similar increases in Glu during motor tasks (Schaller et al. 2014; Volovyk and Tal 2020). Meanwhile, a significant decrease in gamma‐aminobutyric acid (GABA) was reported (Chen et al. 2017). GABA is the main inhibitory neurotransmitter in the brain, however at any timepoint, the majority of GABA in the brain forms a metabolic pool while the minority is neurotransmitter (Rae 2014). Therefore, changes in conventional MRS‐GABA collected at rest, or in a blocked fMRS design likely reflect alterations in tonic rather than phasic inhibition (Rae 2014; Stagg et al. 2011). On the other hand, Glu is the main excitatory neurotransmitter in the brain, and a novel simultaneous fMRS/fMRI experiment has shown that MRS‐Glu and fMRI‐BOLD activation are significantly correlated over time (Ip et al. 2017). This suggests that increases in MRS‐Glu could reflect increases in glutamatergic neuronal firing (Ip et al. 2017), however there remains a degree of speculation regarding the origin of MRS measured signals, including for Glu (Mullins 2018).

fMRS is a powerful approach which allows non‐invasive in vivo quantification of neurometabolites. Recent studies at ultra‐high field (7 T) in the visual (Boillat et al. 2020; Ip et al. 2017) and motor (Chen et al. 2017; Kolasinski et al. 2019) cortex have demonstrated that fMRS can be successfully used to detect task related alterations in the level of metabolites such as Glu and GABA. Performing ultra‐high field MRS offers a higher signal‐to‐noise ratio (SNR) and greater spectral dispersion resulting in improved quantification. As a result, it is possible to detect contributions from peaks which overlap at lower field strengths, such as Glu and Gln, in addition to acquiring higher signal from low concentration metabolites such as GABA (Godlewska et al. 2017). The detection of GABA can be further enhanced using editing approaches which allow unambiguous assignment of GABA in the difference spectrum (Puts and Edden 2012).

In this study we investigated the impact of repetitive MNS on healthy adults as a bridge to enhance our knowledge into the therapeutic potential of MNS. While rhythmic MNS has been shown to influence oscillatory activity, we know little about its effects on neurometabolites, such as GABA and Glu. Given the neurometabolic changes associated with sensorimotor activation during movement (Chen et al. 2017), we hypothesize that repetitive trains of MNS at 10 Hz may lead to an increase in Glu and a decrease in GABA concentration. This study aims to test this hypothesis using ultra‐high field fMRS. As any differences observed in MRS‐GABA or MRS‐Glu could simply be due to a dose‐dependent effect while comparing the effects of rhythmic stimulation to sham stimulation (e.g., 50% of threshold), we have chosen to compare to an arrhythmic control. It is important to note, that rhythmic 10 Hz MNS has previously been demonstrated to be more effective than sham at reducing the frequency of tics in individuals with Tourette syndrome (Maiquez et al. 2023).

## 2 Methods

### 2.1 Participants

Seventeen neurologically healthy and unmedicated adults were recruited for this study. Two were excluded prior to data collection; one due to mild claustrophobia/nausea while in the scanner and the other due to an inability to produce a sufficient muscle twitch at a comfortable MNS intensity. The remaining sample of 15 participants completed two scanning sessions in a counterbalanced order. These sessions were spaced 10 ± 8 days apart and the time of the session was held constant (i.e., if the first session was conducted in the morning, so was the second) for all but one participant due to a change in availability. All participants were deemed right‐handed using the Edinburgh Handedness Inventory (Oldfield 1971). The mean participant age was 27 ± 5 years and 8 were female. Participant demographics can be seen in Table 1. The study received ethical approval through the University of Nottingham School of Psychology committee (Reference number: F1226, Date: August 10, 2020) and all participants gave informed consent.

**TABLE 1:** Participant demographics for rhythmic and arrhythmic conditions.

	*N*	Sex (m/f)	Age (years)	MNS intensity (mA)	Difference in intensity between sessions (mA)
Rhythmic	15	7/8	27.5 ± 4.8	11.3 ± 2.1	0.3 ± 2.3
Arrhythmic	15	7/8	27.5 ± 4.8	11.4 ± 3.0	

*Note*: Counterbalanced within subject design used. Data are presented as mean value ± sd.

### 2.2 MNS Stimulation Paradigm

**FIGURE 1:**
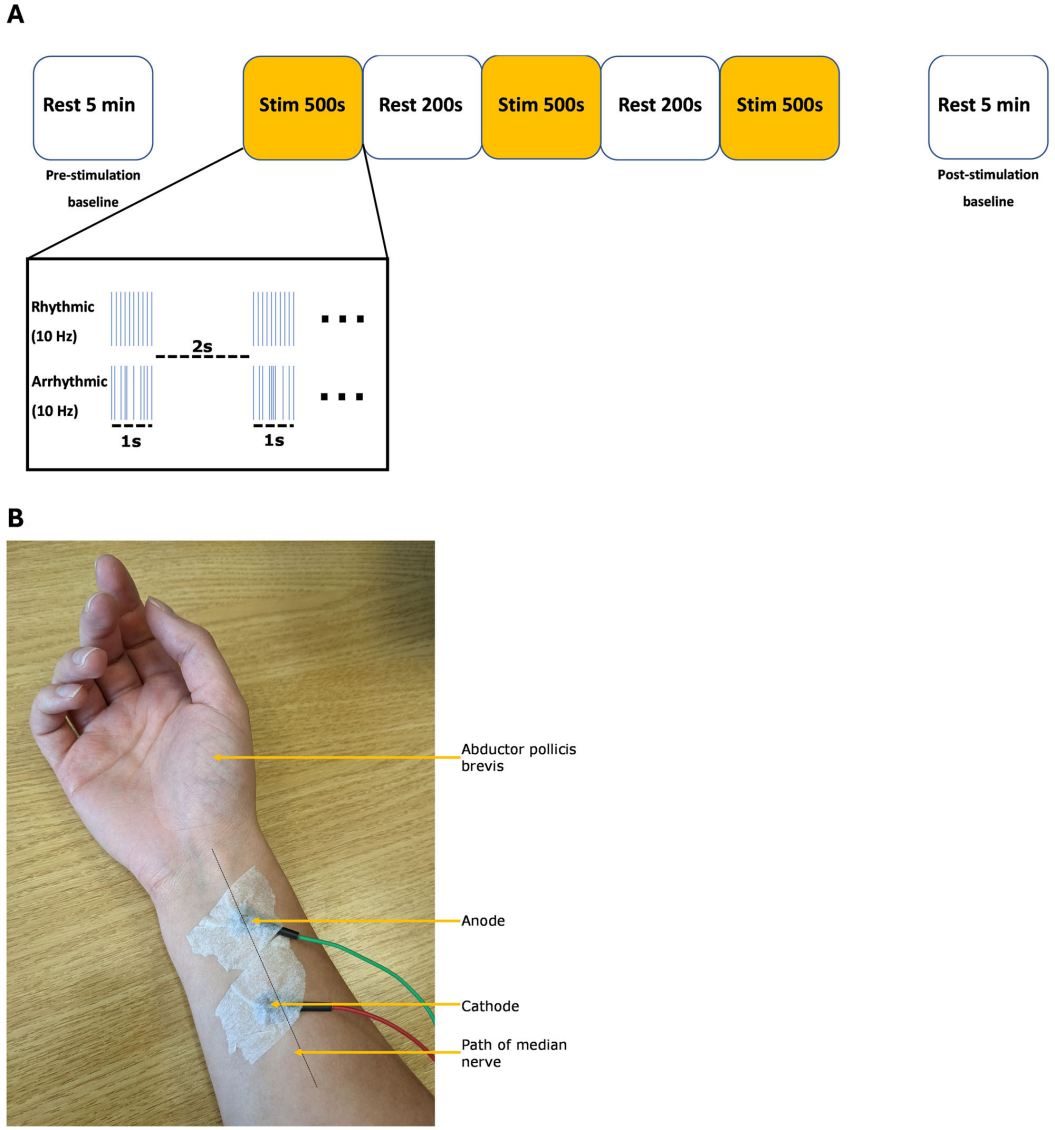
(A) A diagram demonstrating both the trial setup and the stimulation paradigm. (B) A photo showing the electrode placement used.

Stimulation was delivered to the right median nerve using a Digitimer constant current stimulator model DS7A and Ag/AgCl cup electrodes (diameter 10 mm) (Digitimer Ltd, UK) (Figure 1B). The maximum compliance voltage (Vmax) was set to 400 V, and the pulse width was 0.2 ms. The stimulation threshold for each participant was determined to be the minimum intensity which induced a visible thumb twitch (Table 1). During thresholding, participants were asked where they could feel the stimulation to ensure we were targeting the median nerve. During the stimulation blocks, which lasted 500 s, stimulation was delivered at 10 Hz for 1 s followed by 2 s of no stimulation. Stimulation was not delivered constantly for the 500 s to ensure participant comfort. The three stimulation blocks were interspersed with blocks of no stimulation lasting 200 s. Delivery of the stimulation was controlled using MATLAB (MATLAB R2017a, Mathworks, Natick, MA). Each participant completed one session of rhythmic stimulation and one of arrhythmic stimulation. The arrhythmic session was used to investigate whether similar neurometabolic changes occurred when stimulation had a random interpulse interval but the same average frequency (minimum interpulse interval of 0.01 s) (Figure 1A).

### 2.3 MR Acquisitions

MR data were acquired using a Philips 7 T Achieva MR scanner (Philips Healthcare, Best, the Netherlands) with a 32‐channel receiver array head coil. A pair of prism glasses were used to permit the participants to view a nature documentary displayed on a screen outside of the scanner bore for the duration of the scan.

**FIGURE 2:**
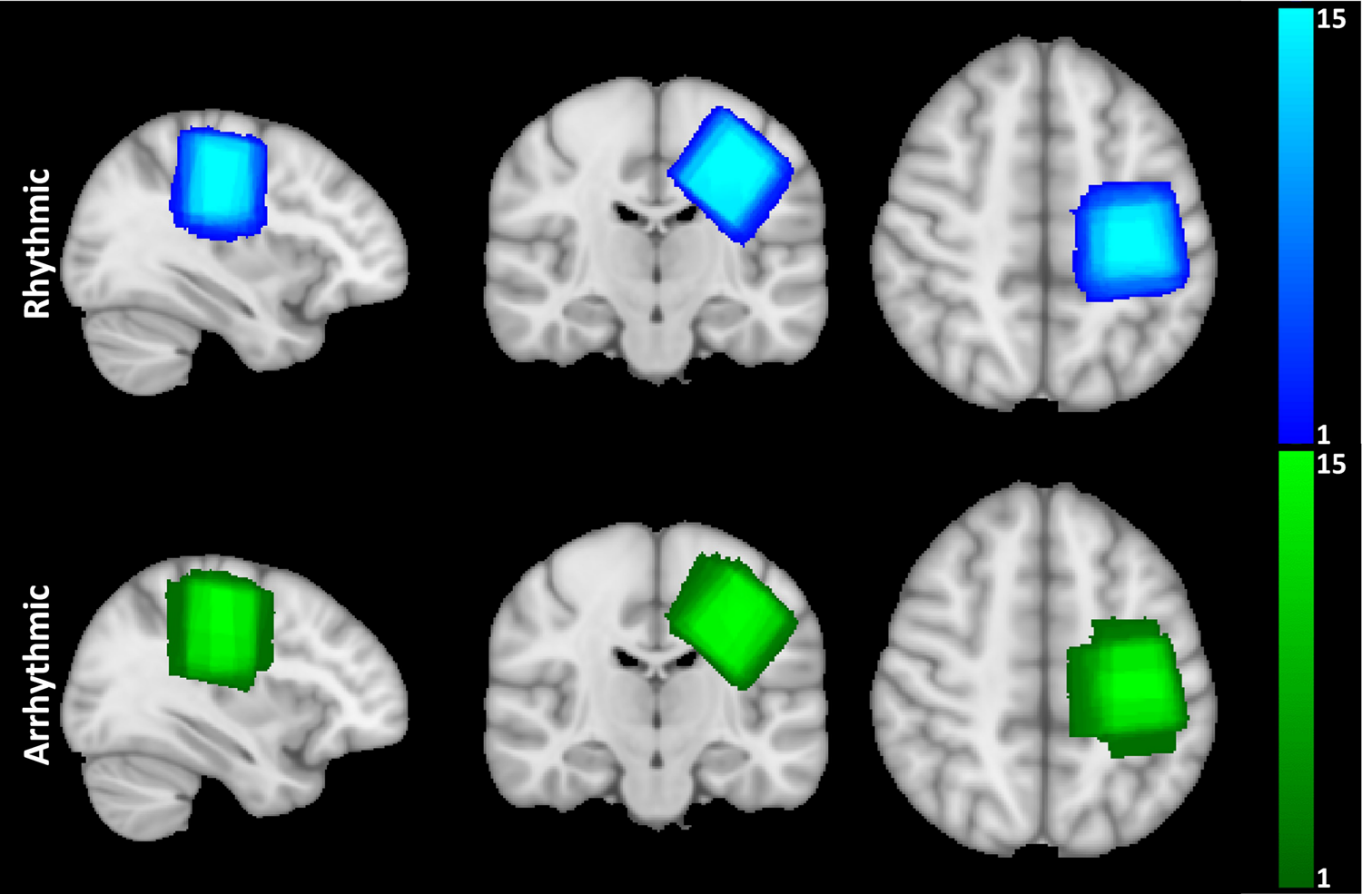
MRS voxel (30 × 30 × 30 mm^3^) overlaps for the rhythmic and arrhythmic sessions centered on the hand knob of the contralateral sensorimotor cortex. Color bars signify the number of subjects.

*T_1_*‐weighted anatomical images were acquired using a MPRAGE sequence (TR/TE/TI = 7.3/3.4/1000 ms, FA = 8°, FOV = 256 × 256 × 180 mm^3^, isotropic resolution = 1 mm^3^) for tissue segmentation (using SPM12) and planning of the MRS voxel. ^1^H MRS data were acquired from a voxel of interest (30 × 30 × 30 mm^3^) placed over the contralateral hand area (Figure 2) using a Mescher–Garwood‐semi‐localized by adiabatic selective refocusing (MEGA‐sLASER) sequence optimized for GABA (TR/TE = 4640/72 ms, spectral width = 4 kHz) (Andreychenko et al. 2012; Mescher et al. 1998). Water suppression was achieved using variable pulse power and optimized relaxation delay (VAPOR) (Tkáč et al. 1999). B_0_‐shimming was performed using a vendor‐provided second‐order projection‐based method. Further methodological details are provided in Supporting Information in accordance with recent recommendations (Lin et al. 2021).

Three consecutive MRS scans were performed during each experimental session. The MEGA‐sLASER parameters were identical for each, except for number of signals averaged and hence total scan time. Pre and post MNS scans occurred without stimulation and lasted approximately 5 min 20 s and consisted of 64 transients consisting of 32 ON/OFF pairs. Scans taken during MNS in both the rhythmic and arrhythmic conditions lasted approximately 32 min 5 s and consisted of a total of 410 transients consisting of 205 ON/OFF pairs. For two participants in one session, the voxel was repositioned following the prestimulation baseline scan due to movement at the beginning of the stimulation block.

### 2.4 Data Analysis

Raw spectral data (.data/.list format) were pre‐processed using an in‐house MATLAB script (MATLAB R2020a, Mathworks, Natick, MA). Raw data were coil‐combined and eddy‐current corrected before being split into ON and OFF spectra. Spectral registration was performed to align individual transients (frequency and phase) to the mean OFF spectra for that participant (Near et al. 2015). Following alignment, individual transients were rejected if the mean square error around the Choline (Cho) peak differed from the mean by more than 3 standard deviations. The aligned ON and OFF spectra were then subtracted to create difference (DIFF) spectra.

For the fMRS acquired during stimulation, spectra were averaged over each block to create a timecourse. Blocks comprised of a spectral average of 108 transients (54 DIFF spectra) for the stimulation periods and 42 transients (21 DIFF spectra) for the rest periods. Each timecourse comprised 5 timepoints. For the prestimulation and poststimulation scans we obtained 1 timepoint for each, consisting of 64 transients.

The GABA DIFF and OFF spectra were fitted in LCModel (Provencher 2001). Spectra were fit with simulated basis spectra from 2D density matrix simulations with shaped refocusing pulse information and inter‐pulse timings (Govindaraju, Young, and Maudsley 2015; Tkáč 2008). The LCModel *nobase* control parameter was set to false to enable baseline fitting. The knot parameter was set to DKNTMN. The spectral range was set to 1.8–4.2 ppm. The total creatine (tCr) and Glu concentrations were quantified using the LCModel concentrations in the OFF spectra, while GABA was quantified using the DIFF spectra. Concentrations are presented as a ratio relative to tCr. A priori exclusion criteria were if the SNR of *N*‐acetylaspartate (NAA) was less than 40, or if the linewidth of unsuppressed water was greater than 15 Hz (0.05 ppm). No participants were excluded by these criteria. The SNR was calculated using the FID‐A MRS toolbox (https://github.com/CIC‐methods/FID‐A) (Simpson et al. 2017). One participant was excluded due to having noisy spectra (Female, 37 years, right‐handed, 11.5 mA intensity for both conditions).

### 2.5 Statistical Analysis

Change ratios were calculated with respect to the prestimulation baseline for that session (Chen et al. 2017). These difference‐from‐baseline measures were then standardized (Z‐transformed) for each participant. Difference‐from‐baseline measures for each block for rhythmic versus arrhythmic were compared using a paired *t*‐test, or a Wilcoxon signed‐rank test where data failed the Kolmogorov–Smirnov test of normality. Difference‐from‐baseline measures for the combined stimulation data for each block were compared using a one‐way ANOVA. The effect size was measured using Cohen's *d*. To examine if difference‐from‐baseline values were modulated by period, a regression (general linear model [GLM]) analysis was conducted with stimulation (i.e., ON vs. OFF) entered as a predictor. An additional GLM analysis was conducted with block (1–6) entered as a predictor, to investigate if there was a linear trend for Glu/tCr difference values to decrease over time.

## 3 Results

**FIGURE 3:**
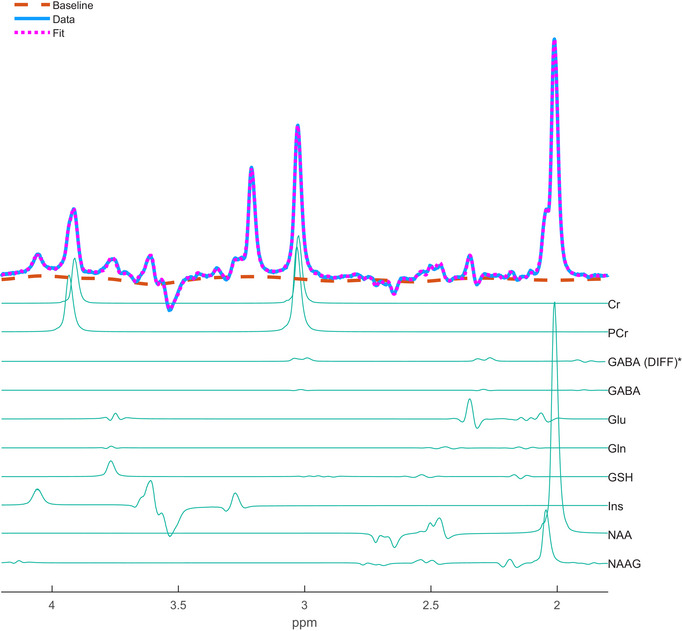
Example MEGA‐sLASER OFF spectra (TE = 72 ms) from an individual subject session showing the average spectra from the fMRS stimulation scan (blue) and the average LCModel fit (pink dashed line). (*GABA concentrations were measured from DIFF spectra).

The data quality metrics of the MRS data including SNR, unsuppressed water linewidth and Cramér–Rao lower bounds (CRLBs) for Glu and GABA can be seen in Table 2. The low linewidth of the water peak implies that good shimming was achieved. Figure 3 shows the quality of both the GABA and Glu LCModel spectra fit at the individual‐level, from a representative subject, during the fMRS stimulation blocks. To check that we could reliably fit Gln from Glu, we ensured that the pair‐wise correlation coefficient for all scans was greater than −0.5.

**TABLE 2:** Data quality metrics for all scans in rhythmic and arrhythmic conditions (*N* = 14).

	Rhythmic baseline	Rhythmic post‐stimulation	Rhythmic stimulation	Arrhythmic baseline	Arrhythmic post‐stimulation	Arrhythmic stimulation
Linewidth of unsuppressed water peak (Hz)	11.1 ± 1.0	11.2 ± 0.9	10.9 ± 0.9	11.0 ± 1.4	11.3 ± 1.2	11.0 ± 1.0
SNR of NAA	281.8 ± 41.2	274.0 ± 35.4	693.7 ± 86.5	273.5 ± 51.5	259.4 ± 47.5	659.3 ± 114.0
GABA CRLB (%)	7.9 ± 2.3	7.9 ± 1.9	7.3 ± 1.3	7.8 ± 1.5	7.6 ± 1.6	7.6 ± 1.9
Glu CRLB (%)	3.4 ± 0.5	3.5 ± 0.5	3.4 ± 0.5	3.4 ± 0.6	3.4 ± 0.5	3.4 ± 0.5

*Note*: Data are presented as mean value ± SD. Abbreviations: CRLB, Cramér–Rao lower bound; GABA, gamma‐aminobutyric acid; Glu, glutamate; NAA, *N*‐acetylaspartate; SNR, signal‐to‐noise ratio.

Figure 2 shows reliable positioning of the MRS voxel over the contralateral hand area for both stimulation sessions. This voxel was composed of 67 ± 3% white matter (WM), 29 ± 3% gray matter (GM), and 4 ± 2% cerebrospinal fluid (CSF). Paired samples *t*‐tests confirmed that there were no significant differences in voxel composition for WM, GM, or CSF between rhythmic and arrhythmic sessions (all *p* > 0.2).

**FIGURE 4:**
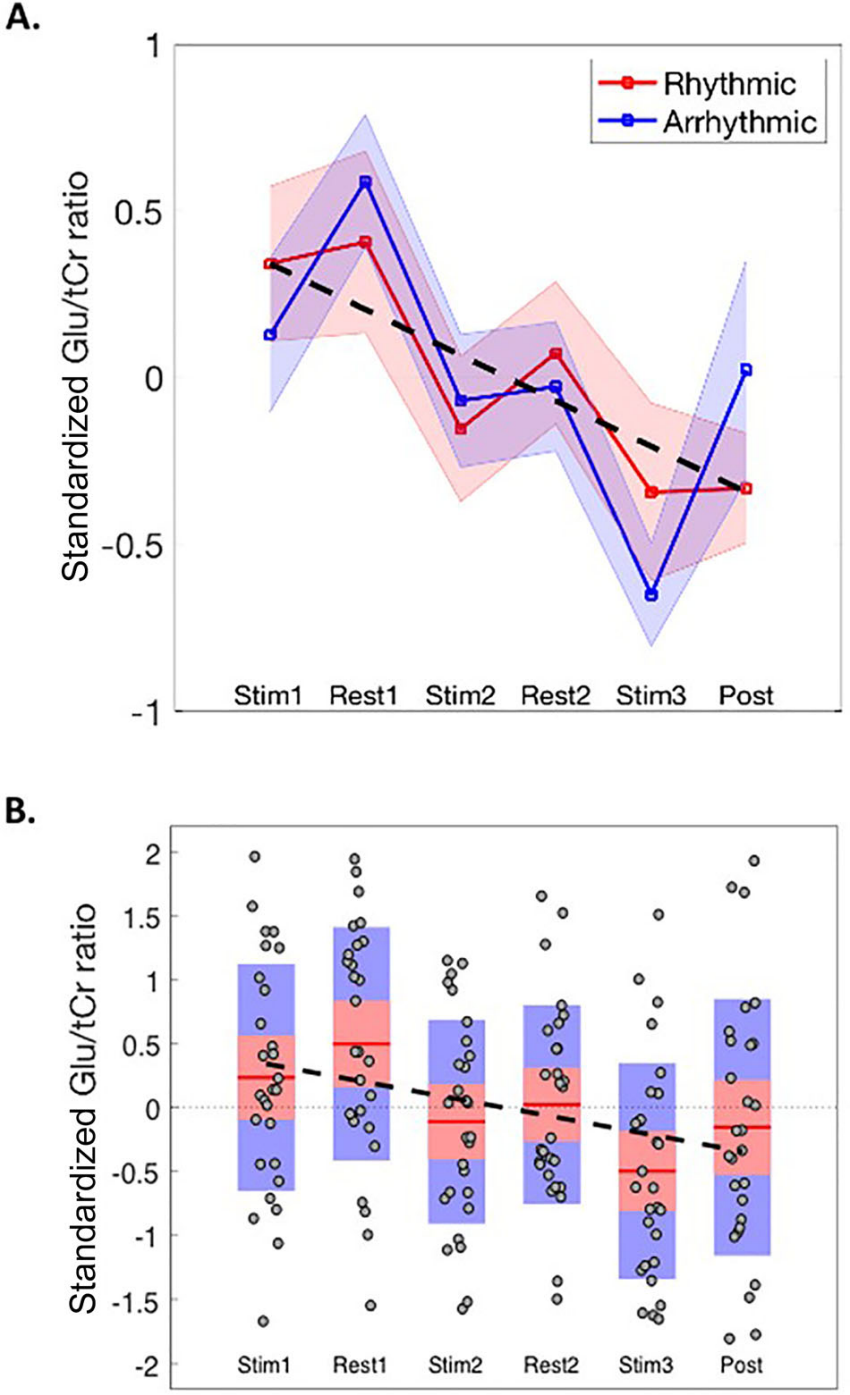
(A) Difference‐from‐baseline measures for Glu/tCr ratio for rhythmic versus arrhythmic stimulation conditions. The shading represents the standard error of the mean (sem). The broken black line illustrates a linear line‐of‐best‐fit for the combined data. (B) Box‐and‐whisker plots of the combined data. Mean values are indicated by the red horizontal lines. The red and blue rectangles represent one standard deviation (red) and the 95% confidence interval (blue). Individual data points are represented by black circles. The broken black line illustrates a linear line‐of‐best‐fit for the combined data.

Initial analyses demonstrated that the difference‐from‐baseline measure for rhythmic compared to arrhythmic stimulation was equivalent for both Glu/tCr (*p* > 0.05) and GABA/tCr (*p* > 0.05) ratios. Relevant means are presented in Figure 4. Therefore, rhythmic and arrhythmic data were combined to explore the effect of stimulation, regardless of pattern, on neurometabolite concentration.

### 3.1 Difference‐From‐Baseline Glu/tCr

Inspection of Figure 4 suggests that Glu/tCr ratio difference values are modulated by stimulation (i.e., ON vs. OFF) and that the magnitude of the Glu/tCr ratio difference values decrease over time. A one‐way ANOVA demonstrated that there was a statistically significant effect of period (*F*_(5,162)_ = 4.3, *p* < 0.002). To examine if Glu/tCr difference values were modulated by period, a regression (GLM) analysis was conducted with stimulation (i.e., ON vs. OFF) entered as a predictor. These analyses demonstrated that the effect of stimulation did not reach conventional levels of statistical significance (*F* = 3.09, Rsq = 0.02, *p* = 0.08). To examine if there was a linear trend for Glu/tCr difference values to decrease over time, a second regression (GLM) analysis was conducted with block (1–6) entered as a predictor. These analyses demonstrated that the effect of block was statistically significant and confirmed that Glu/tCr decreased linearly over time (Slope = −0.14, *F* = 11.7, Rsq = 0.07, *p* < 0.001).

### 3.2 Difference‐From‐Baseline GABA/tCr

**FIGURE 5:**
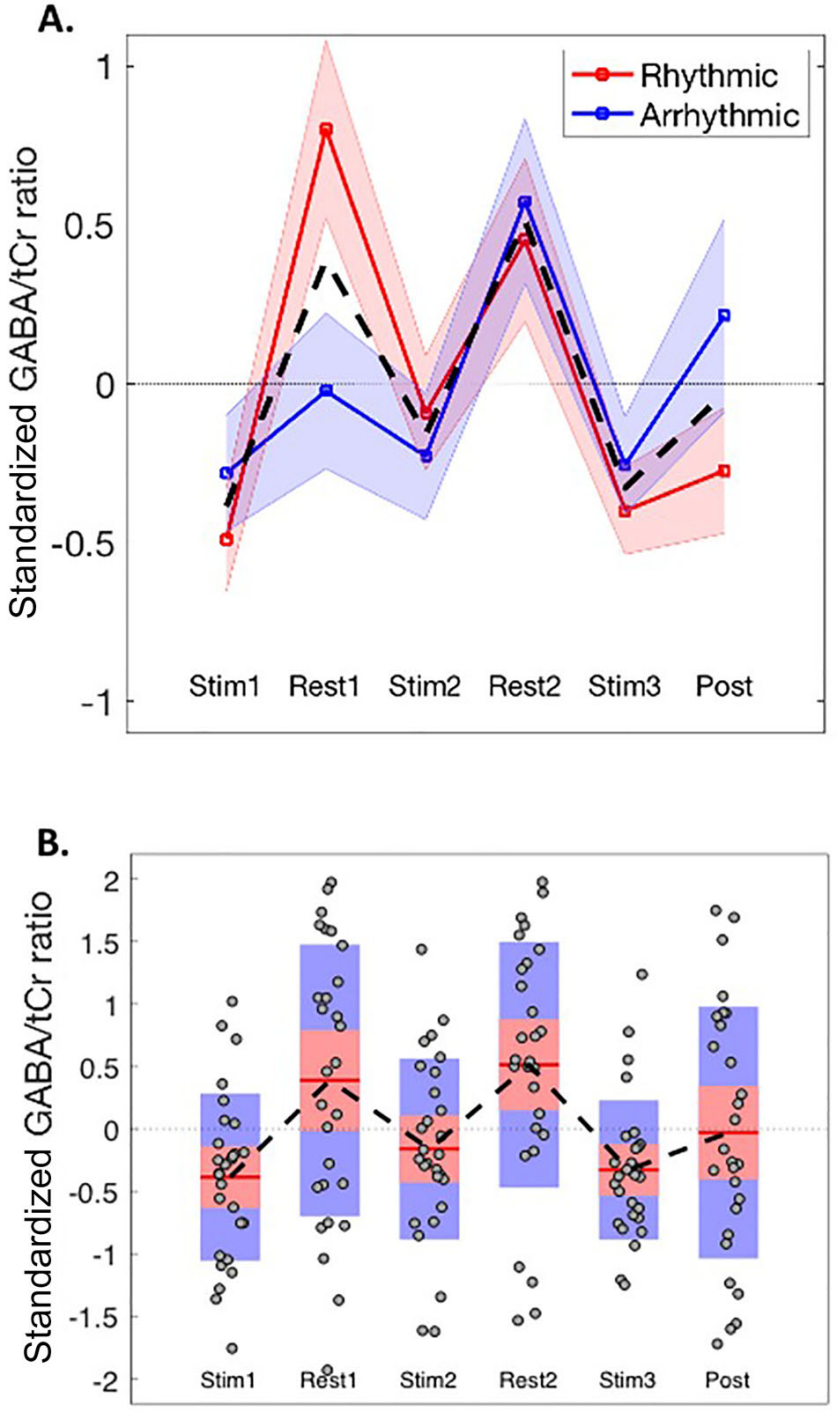
(A) Difference‐from‐baseline measures for GABA/tCr ratio for rhythmic versus arrhythmic stimulation conditions. The shading represents the standard error of the mean (sem). The broken black line illustrates the mean values for the combined data. (B) Box‐and‐whisker plots of the combined data. Mean values are indicated by the red horizontal lines. The red and blue rectangles represent one standard deviation (red) and the 95% confidence interval (blue). Individual data points are represented by black circles.

Inspection of Figure 5 suggests that GABA/tCr ratio difference values are modulated by stimulation (i.e., ON vs. OFF). A one‐way ANOVA demonstrated that there was a statistically significant effect of period (*F*_(5,162)_ = 5.3, *p* = 0.0002). To specifically examine if GABA/tCr difference values were modulated by period, a regression (GLM) analysis was conducted with stimulation (i.e., ON vs. OFF) entered as a predictor. These analyses demonstrated that there was a strong and statistically significant effect of stimulation (*F* = 18.8, Rsq = 0.1, *p* < 0.0001). Additional analyses showed that this effect was present if rhythmic and arrhythmic stimulations were examined separately (minimum *F* = 7.0, *p* < 0.01).

## 4 Discussion

This fMRS study investigated the effects of rhythmic and arrhythmic MNS on neurometabolite concentrations in the contralateral sensorimotor cortex. When overall difference‐from‐baseline measures were compared, there were no statistically significant differences observed between the effects of rhythmic and arrhythmic stimulation on Glu or GABA concentrations. However, when we examined the effects of stimulation, irrespective of stimulation pattern (i.e., rhythmic or arrhythmic stimulation), we do see clear evidence that stimulation modulates concentrations of both Glu and GABA. Specifically, stimulation leads to a linear decrease in Glu concentration over time. By contrast, GABA concentrations decrease during stimulation but increase once again each time stimulation ceases. These effects are discussed below.

Previous studies involving a motor task have also reported increases in Glu (Chen et al. 2017; Schaller et al. 2014; Volovyk and Tal 2020). Our finding of increased Glu during somatosensory stimulation is consistent with the findings reported by Chen et al. (2017), that Glu concentration increases during sensorimotor activity. In addition, our study suggests that stimulation led to a significant reduction in GABA concentration, with higher concentrations seen during rest. These results are also in line with Chen et al. (2017), who reported a decrease in GABA during a sensorimotor hand clenching task. However, such decreases in GABA have not been reliably reported. A study by Volovyk and Tal (2020) investigated alterations in Glu and GABA during a hand clenching task. They reported increases in Glu during hand clenching but no change in GABA. However, it should be noted that this study was conducted at 3 T rather than 7 T. Similarly, a preclinical study demonstrated an increase in Glu but no change in GABA in the contralateral S1 of mice during electrical hind paw stimulation (Seuwen et al. 2019).

The difference between the rhythmic and arrhythmic stimulation conditions is the rhythmicity, or not, of the stimulation. Thus, both forms of stimulation contain an identical number of pulses, of the same duration and intensity, and the mean stimulation frequency is the same. We have previously demonstrated using magnetoencephalography (MEG) that rhythmic MNS increased the power and phase‐synchrony of brain oscillations at the stimulation frequency within the contralateral sensorimotor area, whereas arrhythmic stimulation did not (Houlgreave et al. 2022). Furthermore, in a recent, as yet unpublished, study we found that both rhythmic and arrhythmic MNS delivered to the right wrist leads to a significant increase in fMRI BOLD response in contralateral S1, bilateral secondary somatosensory cortex, and bilateral insula cortex. For both patterns of stimulation, each single MNS pulse will lead to synchronous firing within a population of neurons within the contralateral somatosensory cortex; meaning excited pyramidal cells will be releasing Glu, phasically in synchrony. Importantly, Glu concentrations have been shown to be lower during repetitive trials compared to novel trials (Apšvalka et al. 2015). In the current study we found that Glu levels show an initial increase but then reduce linearly over successive blocks, therefore, Glu may reduce over time in both conditions due to the repetitive nature of the stimulation. This adaptation has not been shown in previous MRS studies involving motor tasks (Chen et al. 2017; Schaller et al. 2014; Volovyk and Tal 2020), but here the stimulation is at a high frequency and externally driven.

Our hypotheses for this study did not consider entrainment effects of the rhythmic stimulation on sensorimotor oscillations (Houlgreave et al. 2022; Morera Maiquez et al. 2020). GABA is thought to be involved in the generation of synchronized oscillations (Gonzalez‐Burgos and Lewis 2008). Previous electrophysiological studies have demonstrated a clear relationship between GABA and oscillations within the sensorimotor cortex. Elevation of the effects of extracellular GABA, using transporter blocker tiagabine and extra‐synaptic positive allosteric modulator gaboxadol, resulted in an increase in the power of all frequency bands up to and including beta oscillations (Nutt et al. 2015). Higher levels of resting MRS‐GABA in the motor cortex have been associated with higher power during the post‐movement beta rebound (Gaetz et al. 2011). Given this relationship between GABA and oscillatory activity, an increase in GABA related to entrainment may have been expected in the rhythmic condition (Spooner, Wiesman, and Wilson 2022). However, the effects of entrainment are, by definition, restricted to the frequency of stimulation. During rhythmic MNS, desynchronization of frequencies within the 8–30 Hz range was evident (Houlgreave et al. 2022; Morera Maiquez et al. 2020). Moreover, any inhibitory effects of entrainment through MNS will be phasic. Phasic inhibition relates to synaptic GABA release leading to short‐lived neuronal hyperpolarization (Brickley and Mody 2012). By contrast, extra‐synaptic GABA binding leads to a more long‐lived tonic inhibition (Brickley and Mody 2012). There is evidence to suggest that there is no association between MRS‐GABA and TMS measures which are thought to reflect activity involving synaptic GABA in adults (Dyke et al. 2017; Stagg et al. 2011; Tremblay et al. 2013). This suggests that MRS‐GABA may be a measure of tonic rather than phasic inhibition. As such, increases in phasic GABAergic activity relating to MNS induced entrainment are unlikely to be quantifiable through MRS. However, it is worth noting that there is evidence for an association between MRS‐GABA and TMS measures in pediatric populations (Harris et al. 2021). In the current study, we clearly demonstrate that GABA levels are substantially decreased during stimulation but are elevated once again during periods of no stimulation. Furthermore, this repeated reduction of GABA levels during stimulation is observed for both rhythmic and arrhythmic stimulation suggesting that any modulation of GABA levels is not frequency specific and is unlikely to be linked to the phasic release of GABA in the form of neurotransmitter.

One limitation of this study is that the stimulation was not constant during the stimulation blocks. Instead, the stimulation was delivered for 1 s followed by 2 s of no stimulation. This choice was made to ensure participant comfort during the experiment. However, data from previous studies shows a gradual return of GABA concentration to baseline following a movement task (Chen et al. 2017), and a gradual increase in GABA concentration during the task even when there were brief pauses between movements (Kolasinski et al. 2019). Another limitation is that the size and therefore SNR of the prestimulation baseline, rest blocks, stimulation blocks, and post‐stimulation blocks were not equivalent. However, the CRLBs for Glu and GABA fitting were not significantly different across all scans. Recent dynamic fitting approaches for MRS, incorporating temporal modeling of the stimulation periods, may offer improved ability to detect changes compared with block‐averaging (Clarke et al. 2024; Tal 2023).

Another limitation of the current study is that we cannot rule out the possibility that the results shown here are due to a startle response. Nevertheless, if the results were associated with a startle response at the start of each block of stimulation, we would expect the effects on both neurometabolite concentrations to decrease during the rest blocks, however, this is not the case for Glu.

## 5 Conclusions

To conclude, this research demonstrates that there is a trend for an initial rise in Glu concentration followed by a decrease over repeated trials, and decreases in GABA during stimulation which recovered during rest, regardless of the pattern of stimulation. Furthermore, the neuromodulatory effects of rhythmic and arrhythmic stimulation were similar despite the entrainment seen previously with rhythmic stimulation (Houlgreave et al. 2022). Recent evidence also suggests that both rhythmic and arrhythmic stimulation may be effective in reducing tics in Tourette syndrome (Iverson, Arbuckle, Song, et al. 2023; Iverson, Arbuckle, Ueda, et al. 2023; Maiquez et al. 2023; Morera Maiquez et al. 2020). We suggested previously that if both rhythmic and arrhythmic stimulation were effective in reducing tic frequency in Tourette syndrome, then the beneficial effects might be due to a sustained decrease in sensorimotor noise after stimulation, due to the synchronous firing of activated neuronal populations associated with each pulse of stimulation (Houlgreave et al. 2022). This might be accompanied by alterations in tonic levels of neurometabolite concentrations. Here we show a reduction in Glu over time, which may reflect a decrease in glutamatergic firing due to adaptation to the continuous stimulation (Apšvalka et al. 2015; Ip et al. 2017). This may make it less likely that an involuntary movement is generated during continuous stimulation. As an offline reduction in tic severity has been reported following 4‐weeks of at‐home rhythmic MNS using a wearable device (Maiquez et al. 2023), it would be interesting to explore the offline changes in neurometabolites following prolonged periods of stimulation.

## Author Contributions

**Mairi S. Houlgreave**: conceptualization, formal analysis, investigation, methodology, writing–original draft. **Katherine Dyke**: conceptualization, investigation, methodology, writing–original draft, writing–review and editing. **Adam Berrington**: formal analysis, investigation, methodology, writing–review and editing. **Stephen Jackson**: conceptualization, formal analysis, funding acquisition, methodology, supervision, writing–review and editing.

## Ethics Statement

The study received ethical approval through the University of Nottingham School of Psychology committee (Reference number: F1226, Date: August 10, 2020) and all participants gave informed consent.

## Conflicts of Interest

The authors declare no conflicts of interest.

### Peer Review

The peer review history for this article is available at https://publons.com/publon/10.1002/brb3.70250.

## Supporting information



Supporting Information.

## Data Availability

Magnetic resonance spectroscopy data can be made available on request if a formal data sharing agreement is in place. The MATLAB code for MNS delivery is available on OSF https://osf.io/c6pwa/.
